# Comparing two models of outpatient specialised palliative care

**DOI:** 10.1186/s12904-021-00727-0

**Published:** 2021-02-18

**Authors:** Elizabeth Rosted, Birgit Aabom, Bibi Hølge-Hazelton, Mette Raunkiær

**Affiliations:** 1 Department of Oncology and Palliative Care, Zealand University Hospital, Sygehusvej 10, 4000 Roskilde, Denmark; 2 Department of Regional Health Research, University of Southern Denmark, J. B. Winsløws Vej 19, 5000 Odense C, Denmark; 3 Department of Research Support, Zealand University Hospital, Munkesøvej 14, 4000 Roskilde, Denmark; 4 REHPA, The Danish Knowledge Centre for Rehabilitation and Palliative Care, Odense University Hospital and University of Southern Denmark, Nyborg, Denmark

## Abstract

**Background:**

Ideally, patients with life-threatening illness who are suffering from multiple symptoms and reduced quality of life should receive palliative care that addresses their specific needs. The many well-defined clinical pathways may not always leave room for a person-centred and individual approach with respect to symptom control, psychosocial and spiritual support, and practical issues. In deciding how to organize outpatient specialist palliative care (SPC), it is relevant to include the perspectives of both patients and families. Thus, the aim of this study was to compare two models for outpatient SPC: first contact between patient, next-of-kin and doctor/nurse in the form of a home visit; and first visit in the hospital setting.

**Method:**

The study was a comparative mixed methods study with follow-up at one and 3 months. It started with a quantitative strand in the form of a 38-item questionnaire. Data were analysed using linear mixed effects models, with maximum likelihood estimation for each outcome variable. The repeated measurements on patient level were modelled by including random intercepts of patients in the mixed model.

**Results:**

In total, 190 participants were enrolled, of whom 102 answered the first questionnaire.

No differences were found between the two SPC interventions when development in satisfaction with care, communication or overall quality of life were compared. At baseline, a significantly higher score for satisfaction was found, in favour of first visit taking place in the hospital setting (65.91 vs. 55.83; *p* = 0.03) measured by FAMCARE-P16, and more patients were satisfied with availability of nurses and their abilities to listen and communicate than of doctors.

**Conclusion:**

Specialist palliative care is in request for many patients in the late phase of their disease. We found no significant differences in satisfaction with care, communication with health professionals or in overall quality of life between the two models. This may imply that access to SPC is more important than the model that is applied, and that a person-centred approach together with time available may matter more than the context. These two factors should be considered when implementing SPC.

## Background

Patients with life-threatening illness often suffer from reduced quality of life (QOL) and multiple symptoms, such as pain, fatigue, and dyspnoea, related to their illness and/or its treatment [1, 2]. Ideally, these patients should receive palliative care that addresses their specific needs. The many well-defined clinical pathways, i.e. the overarching guidelines for medical treatment, care and follow-up, in general ensure that this care is made available, but they may not always leave room for a person-centred and individual approach in respect to symptom control, psychosocial and spiritual support, along with practical issues [3, 4].

In 2019, 10,160 patients in Denmark suffering from cancer and 1133 with other diagnoses were referred to specialist palliative care (SPC). The median survival from first contact with the team was only 38 days for the cancer patients and 37 days for patients suffering from other diagnoses [5]. Referral to SPC thus occurs late in life and, for some patients, this leaves health professionals with little time to arrange and evaluate care, which is why it is important to learn from other patients’ and carers’ experiences when developing practice. The Danish Health Authority defines palliative care in line with the World Health Organization’s definition [6], as described in Table 1.

**Table 1:** Understanding of Generalist and Specialist Palliative Care

In Denmark, the World Health Organization’ definition of palliative care is used by the Danish Health Authority. Its is an approach that “improves the quality of life of patients and their ffamilies facing the problems associated with life-threatening illness...”. It must “be available in all care settings for patients with moderate to high complexity of need, and is provided by health and social professionals who specialise in palliative care and work within a multi-professional specialist palliative care team” [6]. Specialist palliative care differs from generalist palliative care that is provided for patients and their families with low to moderate complexity of palliative care need. Generalist palliative care is defined as “care provided to those affected by life-threatening as an integral part of standard clinical practice provided by any healthcare professional who is not part of a specialist palliative care team” [6].	

In Denmark, the hospitals are responsible for SPC. The care may be organised as either inpatient hospitalisation or at outpatient clinics. Inpatient care in this context refers to care provided to hospitalised patients by an SPC consultation service. Outpatient refers to care provided to non-hospitalised patients who either visit a clinic at the hospital or receive home visits by the SPC consultant team from the hospital SPC consultation service.

Two recently published meta-analyses, including both inpatient and outpatient SPC, showed that interventions were associated with improvements in patient QOL [7, 8], while only one found a positive result for symptom management [8]. Of the 43 included studies in the latter meta-analysis, satisfaction with care was assessed in 11 trials and, of those, seven reported a significant improvement in satisfaction among SPC recipients [8]. It is not clear whether the type of intervention – inpatient or outpatient – was of importance.

From the patient perspective, personalised care provided by inter-professional teams and communication are key elements of the experience of quality of care and satisfaction [9–11]. Depending on their illness, however, patients may have divergent palliative care needs, which the SPC team should be in a position to address. Personalised palliative care should be tailored to provide the right level of intervention for the right patient in the right setting at the right time [11].

In an interview study, Wentlandt et al. (2016) identified six themes of importance in relation to quality of care and satisfaction in palliative care units. Both patients and families emphasized the importance of an interdisciplinary team for the management of SPC, i.e. doctor, nurse, physiotherapist, nutritional therapist, social worker, psychologist and priest; that the care is attentive and personalised; and that it is family-centred. In addition, the authors found that SPC should be allocated appropriate resources and adequate staffing to provide consistent care, and that the setting should be non-institutionalized, thereby allowing for both privacy and socialization [10]. Communication was the most prevalent theme, which encompassed the following: the SPC team should address expectations and explain care goals, keep patients and families informed about the patient’s condition, listen actively to validate patients’ concerns and individual needs, and provide a safe space for conversations about death and dying [10]. These results are consistent with the qualitative results of a systematic review of patients’, families’, and staff’s satisfaction with end-of-life care [12]. In regard to the intervention studies included in the review, Dy et al. (2008) found that palliative care and hospice teams improve patient and family satisfaction. However, it was also found that many studies does not include satisfaction as an outcome, and that more focus on satisfaction-based elements might improve the effectiveness of end-of-life care interventions and their evaluation [12].

While many researchers have studied in-hospital SPC, much fewer have investigated outpatient SPC in connection with home visits [13, 14]. As a result of this, many published studies does not include standardized outpatient SPC- models. For this reason, it can be problematic to compare interventions, organization or clinical results [13]. In a review study, Hui et al. (2018) found that studies involving interdisciplinary SPC teams were more likely to have a positive outcome than those involving solely nurse-led palliative care, though no single study directly compared interdisciplinary teams with single-practitioner-led models [11]. A review from 2013 found only four well-designed RCTs, but a growing body of non-randomized studies that indicated that outpatient SPC may: improve patient satisfaction, symptom control and QOL; reduce health care utilization; and lengthen survival in a lung cancer population [13]. In addition, only four studies concerning outpatient SPC were found by Zimmermann et al. (2008). Three of those showed significantly improved satisfaction when receiving SPC [15].

This seems to show that both inpatient and outpatient SPC improves patient satisfaction, but it is not clear how the outpatient SPC were organized, as the studies do not describe a standardized outpatient SPC model. However, it is clear that core elements of quality of care and satisfaction are personalised care by inter-professional teams and the teams’ ability to communicate open-minded [9, 10, 16]. This is an individual approach to symptom control, psychosocial and spiritual support, together with practical issues.

In Denmark, most patients are very satisfied with the SPC they receive, however the factors of age, marital status and place of care were found to have some impact, in a nationwide survey from 2012 [17]. In other studies that examined the effect on early SPC no difference were found between intervention and standard care [18, 19].

Although several issues may be common to patients and families, studies show that factors important to quality at the end of life differ by role and by individual [17, 20, 21]. Models may vary in relation to different national health and welfare systems, geography, culture and traditions, and the literature does not clearly prescribe a model for outpatient SPC [22]. Thus, when deciding how to organize outpatient SPC in the future, it is relevant to study outpatient SPC and include both patients’ and families’ perspectives.

Thus, the overall aim of this study was to compare two different models for outpatient SPC, i.e. first contact between patient, next-of-kin and doctor/nurse in the form of a home visit, and the first visit taking place in the hospital setting. This was with a view to gaining new knowledge about satisfaction, symptom control and QOL in relation to outpatient SPC and to include this knowledge in future service planning and organization.

## Method

### Design

The study was a comparative mixed methods study with an explanatory sequential design. It started with a quantitative strand, in the form of a questionnaire, through which general trends and relationships were examined. Results from the quantitative analysis then determined which results needed further exploration in the form of in-depth personal perspectives and what questions to ask participants in the qualitative interview phase [23]. This paper presents results from the quantitative strand – a 38-item questionnaire.

### Participants and setting

The setting was the outpatient SPC units at the Department of Oncology and Palliative Care at Zealand University Hospital, Denmark. In January 2016, the oncological and palliative care departments at five local hospitals in the area of Region Zealand had been merged into one overarching department. The newly re-organized outpatient SPC unit was to have two sections – one located at two hospitals, Roskilde and Koege (R/K) and the other section at three hospitals, Naestved, Slagelse and Nykoebing F (N/S/N). Outpatient SPC was organized differently at the two sections, which offered the opportunity to compare the two models. In 2017, 371 patients were treated at the outpatient sections R/K and 667 at N/S/N [24]. The median length of time from patients’ admission to death was about 3 months. Two of the hospitals associated with the outpatient SPC teams could offer admission to an inpatient SPC unit, if it were required for a patient’s care. Other SPC options in the region were three independent hospices, with a total capacity of 682 admitted patients in 2017 [24].

All adult patients registered to one of the two outpatient SPC sections during 2017 received written information about the study at their first consultation. Those who consented received a telephone call from the researcher within the following week informing them about the study and asking them to complete the questionnaire. Those who consented were included and returned a written consent. Patients were excluded if they did not understand or speak Danish, or if they were considered to be unable to participate due to physical, mental or social reasons, as assessed by the SPC nurses at the first consultation, based on their clinical knowledge.

### The specialised palliative care interventions

The overall purpose of the SPC teams work at Zealand University Hospital is defined by the Danish Health Authority in line with the World Health Organization’s definition of SPC [6, 25].

The primary differences between how the two teams in our study worked were how the first contact was made and the procedure for follow-up. At R/K, the first consultation took place in the hospital setting and follow-up consisted of SPC team consultations every 3 weeks and additional follow-up telephone calls by the SPC nurse. At N/S/N, the first visit by the SPC team was a home visit, and follow-up was conducted primarily by the patient’s general practitioner (GP) and the community home care nurse, who was a non-specialist palliative care nurse. Table 2 shows the two different interventions.

**Table 2:** Specialised Palliative Care interventions at the two study locations

Outpatient clinics	R/K SPC team	N/S/N SPC team
**Capacity**	180	280
**The team receive referrals from:**	Hospital physician Private practitioners Other professionals at the hospital Professionals at hospices Family doctor Home care nurse Professionals at nursing homes and community day centre Patients themselves Patients’ family members	Hospital physician Private practitioners Professionals at hospices Family doctor
**Pre-admission assessment for the SPC teams**	Pre-admission assessment is carried out daily and patients in need of specialised palliative care, regardless of their diagnoses or age, are accepted	Preadmission assessment is carried out daily and patients in need of specialised palliative care, regardless of their diagnoses or age, are accepted
**SPC team staff**	Palliative care physicians Palliative care nurses Physiotherapist Social worker Priest Dietician Psychologist	Palliative care physicians Palliative care nurses Physiotherapist Social worker Priest
**Location of outpatient consultations**	Primarily at the outpatient clinic located at the hospital Home visits	Always home visits
**Duration of association with the SPC team**	From pre-admission assessment to end of life	From pre-admission assessment to well established palliative care, as agreed by patient, family and health professionals
**Hotline service for patients**	Two hours during daytime on weekdays, provided by palliative care nurses	During daytime on weekdays, provided by palliative care nurses
**Hotline service for professionals**	24-hotline service, provided by palliative care physicians and nurses. Offered to professionals from hospitals, nursing homes, family doctors, primary healthcare nurses and other who may call.	During the daytime on weekdays, provided by palliative care physicians and nurses. Offered to professionals from hospitals and nursing homes
**Routine offer of care to family**	Yes	Yes
**Routine offer of care to bereaved family**	Yes	No
**Approach to care**	Multidisciplinary, addressing physical, psychological, social and spiritual needs	Multidisciplinary, addressing physical, psychological, social and spiritual needs

#### Outpatient SPC team at R/K

The SPC team at R/K is interdisciplinary and consists of palliative care physicians and nurses, a physiotherapist, a dietician, a social worker, a psychologist and a priest. The team holds weekly interdisciplinary meetings. The core intervention was consultation and follow-up in the palliative care outpatient clinic at the hospital by a palliative care physician and nurse, consisting of comprehensive, multidisciplinary assessment of symptoms, psychological distress, social support and home services. The first consultation lasted about 1 hour. Follow-up consultations were scheduled every 3 weeks or as required, either in the form of a consultation with the palliative care physician and nurse or with the nurse alone. The follow-up consultation was scheduled to last half an hour. In addition, telephone follow-up by an SPC nurse was offered on a regular basis. If the patient was too ill to consult the team at the hospital, a home visit could be arranged. A telephone hotline for urgent issues was available for 2 hours daily. If patients had SPC needs, they were offered follow-up until the end of life. Ancillary interventions, depending on the status of the patient, included arrangement of community home care nursing, by a non-specialist palliative care nurse, referral to one of three hospices, or admission to the inpatient SPC unit at Zealand University Hospital for urgent symptom control or terminal care.

#### Outpatient SPC team at N/S/N

The SPC team at N/S/N was also interdisciplinary, but had a different staff make-up: palliative care physicians and nurses, a physiotherapist, a social worker and a priest. Weekly interdisciplinary meetings were held. The core intervention was a home visit consultation by a palliative care physician and nurse, consisting of a comprehensive, multidisciplinary assessment of symptoms, psychological distress, social support and home services, with follow-up primarily conducted by a palliative care nurse. The first home visit lasted between one and 2 hours. Most patients received one follow-up home consultation, and one or two follow-up phone calls by the SPC nurse, as needed, and then the patient’s GP and home care nurses assumed responsibility for the palliative care. A telephone hotline for urgent issues was available during the daytime. Ancillary interventions, depending on the status of the patient, included: arrangement of community home care nursing service by a non-specialist palliative care nurse, referral to one of the three hospices or admission to the inpatient oncological unit at Zealand University Hospital, or to a general medical ward, for urgent symptom control or terminal care. The SPC team could refer patients to the local hospices, who would then do a pre-admission assessment, before the patients were admitted. The SPC teams and the hospices worked in close partnership, as some SPC doctors also served the hospice and – as is the case in other countries – the hospice was the provider of SPC in the community. Admission to hospices is free of charge in Denmark.

### Data collection

Participants were included from January until December 2017. Questionnaires were sent at inclusion after the first SPC team consultation and after one and 3 months, either by ordinary post in a paper version with a franked reply envelope or by email, as a link to the online survey, in accordance with patients’ choices. The online survey was developed in SurveyXact (“SurveyXact by Ramboll” 2018), a secure data management application that has certified access and encrypted communication. Reminders were sent to those who had not returned or started the questionnaire within 14 days. Two further reminders were sent, respectively, two and 4 weeks after the first reminder.

Primary outcome was participants’ satisfaction with care and communication. To measure satisfaction with care, we used the family satisfaction scale (FAMCARE-P16). It is a 16-question questionnaire measuring satisfaction in relation to information-giving, availability of care, psychological care and physical care. The score ranges from 16 to 80; the higher the scores, the greater satisfaction [26, 27]. The Cancer Patient’s World Questionnaire (CPWQ-com) measured satisfaction with communication [28]. This brief questionnaire consists of seven questions; the score was transferred to a sum rating from zero to 100, and higher scores indicated greater satisfaction [28].

Secondary outcomes were participants’ symptom control, physical and emotional function and quality of life. Those were measured by European Organization for Research and Treatment of Cancer Quality of Life Questionnaire Core 15 - Palliative Care (EORTC QLQ-C15-PAL) [29], which is a 15-item abbreviation of the EORTC QLQ-C30, developed by European Organization for Research and Treatment of Cancer [30]. It comprises a global health-status scale, two multi-item functional scales (physical functioning and emotional functioning), two multi-item symptom scales (fatigue and pain), and five single-item symptom scales (nausea and vomiting, dyspnoea, insomnia, appetite loss and constipation). Responses are measured in 4-level to 7-level Likert scales; 7-level for global health status and 4-level for the remaining scales, which were linearly transformed into a score of zero to 100; higher scores represent best global health and best functional status but worst symptoms’ [31].

Baseline data collected for both groups included participants’ demographic information along with data on age, sex and diagnosis.

### Statistics

Participants’ characteristics are presented as means with standard deviation (SD) and as proportion. The two groups were compared at baseline using χ^2^ test for categorical variables and T-test or Wilcoxon rank sum test for continuous variables. Linear mixed effects models, using maximum likelihood estimation, were applied for each outcome variable, in order to estimate differences in changes of outcome from baseline to 1st and 2nd follow-up, respectively. The fixed part of the models included dummy variables for time of follow-up and treating hospital, and for interaction between time and hospital. Estimation of a fixed effect of treating hospital allowed for baseline differences in baseline measurements. The repeated measurements on patient level were modelled by including random intercepts of patients in the mixed model. Visual inspection of QQ-plots demonstrated deviations from normal distribution of the residuals; therefore bootstrapping methods using 10,000 repetitions were used when estimating the confidence intervals. Stata 16 (StataCorp. 2019. Stata Statistical Software: Release 16. College Station, TX: StataCorp LLC) was used for the statistical analysis.

The FAMCARE scores were calculated as both a total score and an individual item score. Consistent with previous approaches [32], we identified individuals scoring > 64 as generally satisfied, because this cut-off value is associated with rating satisfied on all 16 items. The results for individual FAMCARE items were dichotomized into satisfied (scores > 4) or dissatisfied (scores < 3), because those differences were deemed to be most clinically relevant.

## Results

**Fig. 1:**
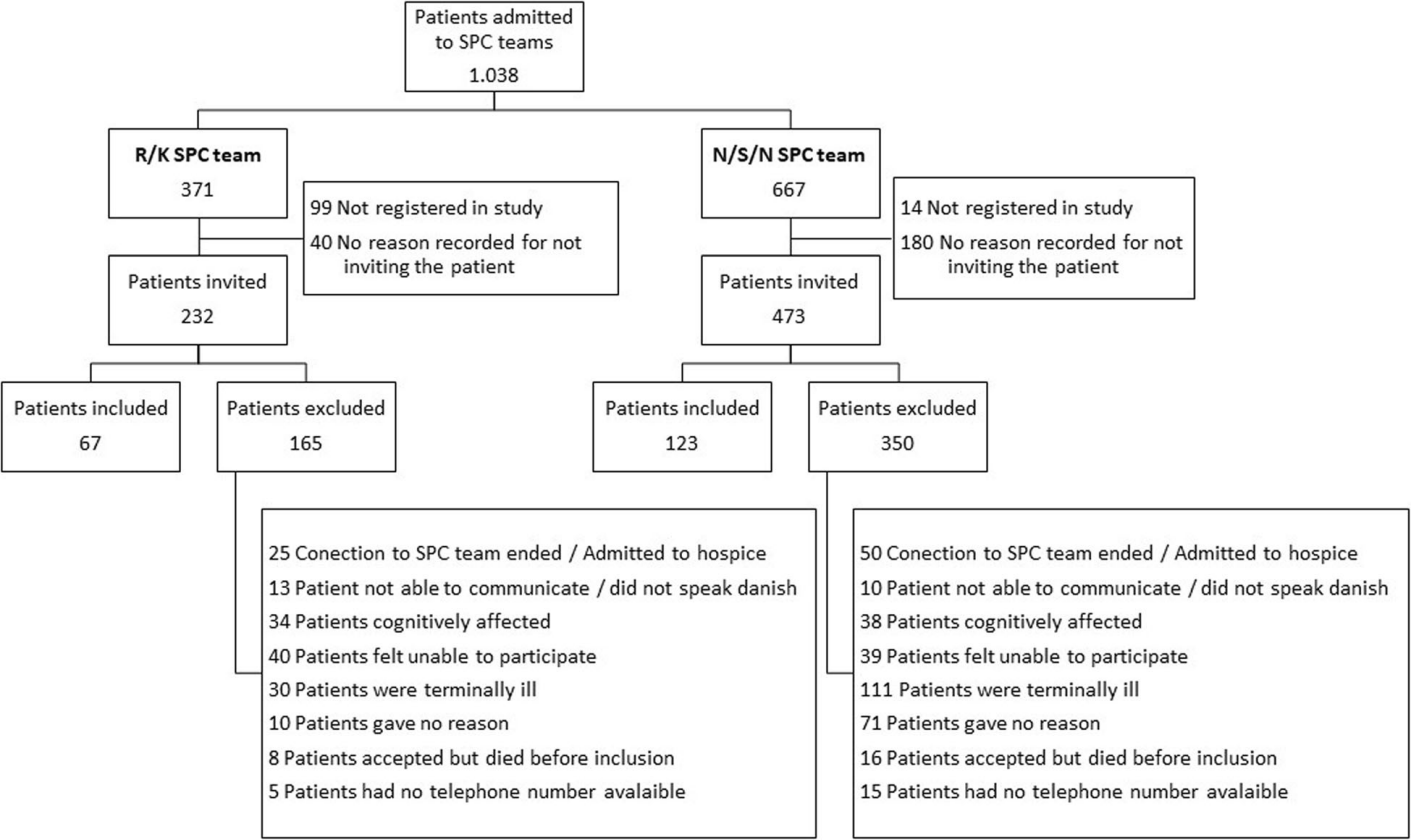
Flow of participants and their reasons for leaving the study

A total of 190 participants were enrolled in the study (67 from R/K and 123 from N/S/N). During 2017, 1038 were registered to the SPC teams (371 to R/K and 667 to N/S/N). A total of 220 eligible patients were listed in the study while 113 were not. The SPC team nurse who was responsible for informing patients about the study and receiving potential participants’ acceptance form was challenged for time. For this reason, including participants for research studies could not be prioritised. A total of 705 were invited to participate and, of those, 190 accepted the invitation. The flow of participants and their reasons for leaving the study is shown in Fig. 1. Of the 190 invited, 102 returned the first questionnaire and were thus included in the study; 55 returned the second questionnaire and 31 the third. Of the 102 the age range was 38 to 88, with a mean age of 68 (SD 9.47). Patients enrolled but not included were the 88 who did not return the first questionnaire. Of those, 80 died within 110 days and the mean residual life was 13 days*.* We found no difference between participants and those not included, with respect to sex, mean age or distribution between the hospitals.

At baseline, the only significant difference in demographic characteristics between the participants from N/R and N/S/N were their level of education, basis for income and distance to hospital, which was expected, given the hospitals’ geographic location (Table 3).

**Table 3:** Patient characteristics at baseline, *n* = 102

	R/K		N/S/N		***p*** value
	Mean (SD)	n (%)	Mean (SD)	n (%)	
Age	70 (9.1)		67 (9.6)		0.13
Sex					0.48
Female		19 (55.9)		43 (63.2)	
Male		15 (44.1)		25 (36.8)	
Marital status, *n* = 101					0.69
Married/Cohabiting		25 (73.5)		46 (68.7)	
Widowed		7 (20.6)		13 (19.4)	
Divorced		2 (5.9)		4 (6.0)	
Living alone		0 (0)		4 (6.0)	
Children living at home, *n* = 101					0.43
Yes		3 (8.8)		3 (4.5)	
No		31 (91.2)		64 (95.5)	
Education, *n* = 94					0.02
Primary/Secondary schooling		6 (18.2)		16 (26.2)	
Vocational training, less than 1 year		2 (6.1)		1 (1.6)	
Vocational training, more than 1 year		2 (6.0)		11 (18.0)	
Diploma		6 (18.2)		17 (27.9)	
Bachelor		11 (33.3)		14 (23.0)	
Master’s degree or more		6 (18.2)		2 (3.3)	
Income based on, *n* = 101					0.01
Work		3 (8.8)		11 (16.4)	
Sickness benefits		0 (0)		2 (3.0)	
Early retirement		1 (2.9)		6 (9.0)	
Pension		26 (76.5)		48 (71.6)	
Self-employed		1 (2.9)		0 (0)	
Other		3 (8.8)		0 (0)	
	Mean (SD)		Mean (SD)		
Distance to hospital, km	11.0 (7.0)		33.4 (20.6)		0.00
Disease site, *n* = 98					0.20
Cancer origin					
Gastro-intestine		8 (24.2)		19 (29.2)	
Lung		8 (24.2)		21 (32.3)	
Breast/female genitals		5 (15.2)		12 (18.5)	
Prostate		2 (6.1)		1 (1.5)	
Other		8 (24.2)		10 (15.4)	
Non-cancer		2 (6.1)		2 (3.1)	
Gamma-test for comparing ordinal data Two sample t-test for comparing mean					

At baseline, patients from hospitals R/K had a significantly higher overall mean score for satisfaction with care (65.91 vs. 55.83; *p* = 0.03), measured by FAMCARE-P16. Based on the individual score of > 64 as generally satisfied, patients from R/N were satisfied, while patients from N/S/N were not satisfied, although in comparing each individual question, we found no significant differences between the two groups. However, more patients were satisfied with the availability of nurses as compared to that of doctors (Table 4).

**Table 4:** Comparison of satisfaction with care between groups at baseline

	R/K		N/S/N		***p*** value	Persons R
	*n* = 24		*n* = 42			
**FAMCARE-P16 score**	Mean (SD)		Mean (SD)			
Total FAMCARE-P16 score, *n* = 66	65.91 (32.17)		55.83 (29.60)		0.03	
*Question:*		Satisfied** n (%)		Satisfied** n (%)	*p* value***	
Information provided about prognosis		17 (70.8)		30 (71.4)	0.96	−0.006
Answers from health professionals		20 (83.3)		36 (85.7)	0.80	−0.032
Information given about side effects		17 (70.8)		30 (71.4)	0.96	−0.006
Referrals to specialists		16 (66.7)		27 (64.3)	0.85	0.024
Speed with which symptoms are treated		19 (79.2)		32 (76.2)	0.79	0.034
Doctor’s attention to description of symptoms		18 (75.0)		24 (57.1)	0.15	0.179
The way tests and treatments are performed		20 (83.3)		35 (83.3)	1.00	0.000
Availability of doctors to answer questions		18 (75.0)		26 (61.9)	0.26	0.134
Availability of nurses to answer questions		23 (95.8)		39 (92.9)	0.63	0.060
Coordination of care		19 (79.2)		32 (76.2)	0.79	0.034
Family inclusion in treatment/care decisions		18 (75.0)		29 (69.0)	0.61	0.063
Information given about how to manage pain		20 (83.3)		28 (66.7)	0.15	0.180
Information given about tests		18 (75.0)		27 (64.3)	0.38	0.111
How thoroughly doctor assesses symptoms		18 (75.0)		25 (59.5)	0.21	0.156
Follow-up on tests and treatments		19 (79.2)		26 (61.9)	0.15	0.178
Availability of doctors to the family		18 (75.0)		22 (52.4)	0.07	0.223
**CPWQ-com score**	*n* = 25		*n* = 48			
	Mean (SD)		Mean (SD)			
CPWQ-com, *n* = 73	76.43 (33.11)		73.75 (19.10)		0.52	
*Question:*		Satisfied** n (%)		Satisfied** n (%)	*p* value***	
Have the doctors been good at communicating?		21 (84.0)		38 (79.2)	0.62	0.058
Have the doctors used understandable language?		22 (88.0)		40 (83.3)	0.60	0.062
Have the doctors been good at listening to you?		22 (88.0)		40 (83.3)	0.60	0.062
Have the nurses been good at communicating?		24 (96.0)		47 (97.9)	0.64	−0.056
Have the nurses been good at listening to you?		24 (96.0)		46 (95.8)	0.97	0.004
Has information been provided at the appropriate time?		20 (87.0)		42 (87.5)	0.95	−0.008
Have the staff allowed enough time for consultations?		21 (87.5)		44 (91.7)	0.58	−0.066
*Independent t-test for comparing mean – equal variances assumed **scoring 1 “very satisfied” or 2 “satisfied” *** Persons R						

Satisfaction with communication, measured by CPWQ-com, was equal in the two groups at baseline. More patients were satisfied with nurses’ abilities to listen and communicate than doctors. In terms of QOL, measured by EORTC QLQ-C15-PAL, we found that patients from hospitals R/K had a significantly higher mean score in relation to physical function (66.99 vs. 58.01; *p* = 0.01). No other differences were found (Table 5).

**Table 5:** Comparison of symptom control, physical and emotional function and quality of life at baseline

	R/K	N/S/N	
**EORTC QLQ-C15-PAL**	*n* = 34	*n* = 68	
	Mean (SD)	Mean (SD)	p value
*Functional scale:*			
Physical functioning	66.99 (20.38)	58.01 (29.48)	0.01
Emotional functioning	69.61 (22.27)	73.00 (26.25)	0.36
*Symptom scale:*			
Fatigue	54.90 (25.14)	56.62 (24.63)	0.74
Nausea and vomiting	20.59 (24.64)	27.36 (34.29)	0.23
Pain	45.10 (31.92)	49.26 (32.89)	0.53
Dyspnoea	28.43 (33.97)	33.82 (32.83)	0.46
Insomnia	26.47 (28.16)	33.82 (31.80)	0.21
Appetite loss	45.10 (30.58)	39.30 (32.27)	0.38
Constipation	15.69 (27.51)	23.38 (29.60)	0.18
*Global Health:*			
QLQ	43.43 (22.41)	50.51 (23.56)	0.10

Comparing differences in satisfaction from baseline to one and 3 months, we found no differences in development of either FAMCARE-P16 or CPWQ-com between the two hospitals.

**Fig. 2:**
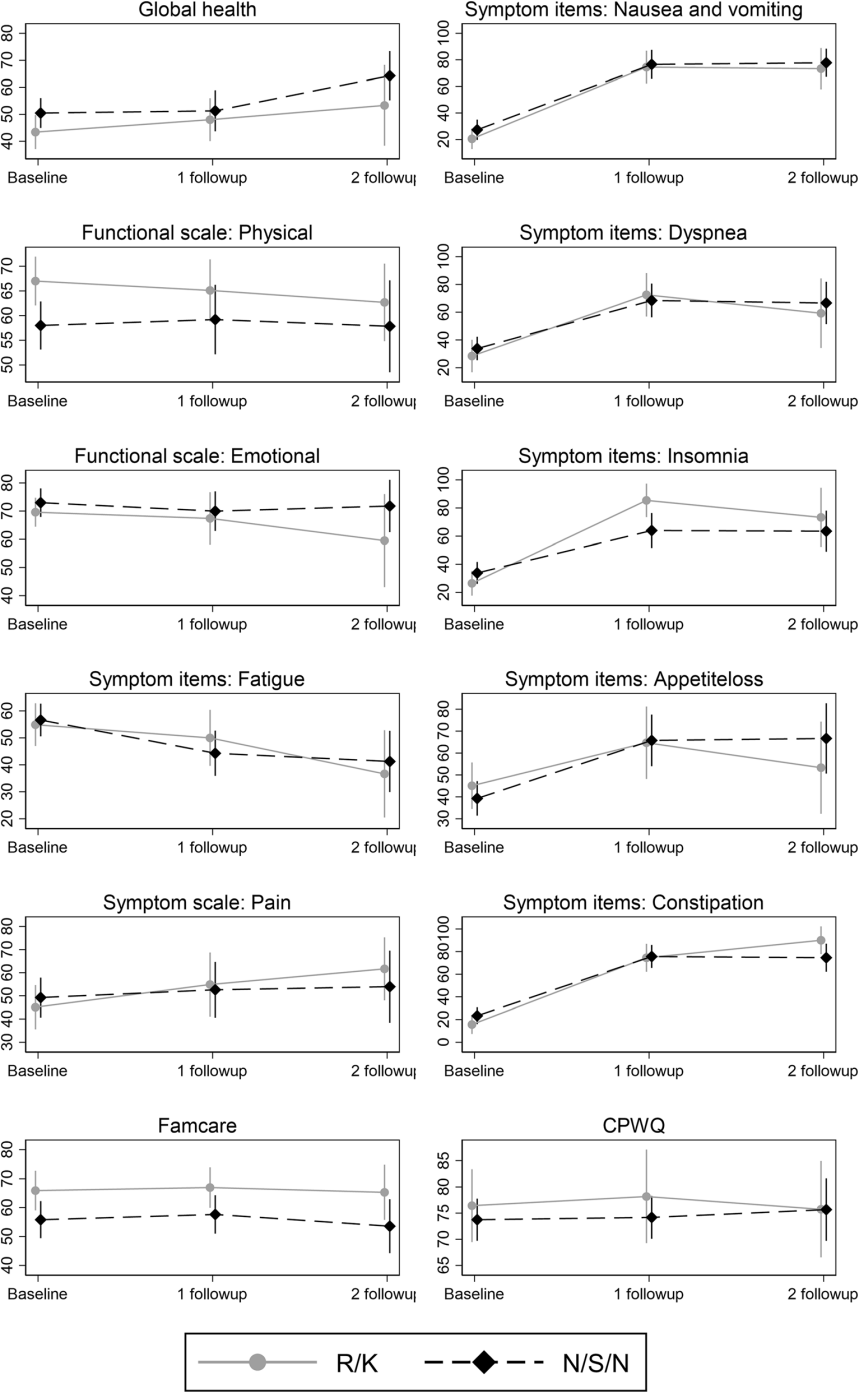
Development in QOL, symptoms, satisfaction and communication

The development in overall QOL did not differ between the two hospitals at one or 3 months. However, in relation to the symptom insomnia, the mean difference in scores were significantly higher *p* = 0.01 at 1 month follow-up for patients at hospital R/K (mean change − 28.81; SE 11.05). In addition, the development in the symptom constipation was significant (*p* = 0.04). The mean change was higher for patients from R/K at 3 months follow-up (mean change − 23.09; SE 11.25) (Table 6 and Fig. 2).

**Table 6:** Change between groups in satisfaction, physical and emotional function, symptom control, and quality of life

	Differences in change from baseline to 1st follow-up		Differences in change from baseline to 2nd follow-up	
	Mean (SE)	*P*	Mean (SE)	*p*
FAMCARE-P16	0.78 (7.33)^b^	0.92	−1.66 (9.07)^a^	0.86
CPWQ-com	−1.30 (7.01)^a^	0.85	2.63 (7.45)^b^	0.74
EORTC QLQ-C15-PAL				
*Functional scale:*				
Physical functioning	3.08 (5.86)^b^	0.60	4.15 (6.77)^b^	0.54
Emotional functioning	−0.82 (7.15)^a^	0.91	8.89 (10.65)^b^	0.40
*Symptom scale:*				
Fatigue	−7.42 (8.92)^a^	0.41	2.89 (11.72)^b^	0.81
Nausea and vomiting	−4.71 (10.73)^a^	0.66	−2.33 (11.47)^a^	0.84
Pain	−6.44 (12.10)^a^	0.60	−11.87 (12.76)^a^	0.35
Dyspnoea	−9.47 (13.04)^a^	0.47	2.02 (17.22)^b^	0.91
Insomnia	−28.81 (11.045)^a^	0.01	−17.19 (14.42)^a^	0.23
Appetite loss	6.85 (13.18)^b^	0.60	19.13 (15.49)^b^	0.22
Constipation	−6.53 (10.32)^a^	0.53	−23.09 (11.25)^a^	0.04
*Global Health:*				
QLQ	−3.79 (7.34)^a^	0.61	3.88 (9.97)^b^	0.70

^a^A negative number indicates that R/K’s patients’ symptoms are increasing or QOL score are decreasing more relative to N/S/N’s patients

^b^A positive number indicates that R/K’s patients’ symptoms are decreasing or QOL score are increasing more relative to N/S/N’s patients

## Discussion

In this study, we found no differences over time between the two models of SPC consultations in terms of overall satisfaction, satisfaction with communication or overall QOL. We found a significant difference in the development of the symptom insomnia at 1 month and in constipation at 3 months. At baseline, there were no significant differences in the symptom insomnia between the two groups that scored in mean 26 (R/K) and 34 (N/S/N). But, at the 1 month follow-up, patients from both groups reported much higher mean scores, i.e. 85 (R/K) and 64 (N/S/N), reflecting a worsening of their insomnia, which was more severe at hospital R/K. In comparison, Nordic studies found a mean score of 24 in a nationally representative sample of advanced cancer patients in Denmark [2] and 39-41in a regional sample of cancer patients in Norway admitted to palliative care [33]. SPC involves the sub-group of patients who are most burdened by symptoms and problems; and as SPC is initiated at the end-stage of life, progression of the disease and symptoms is often rapid [2]. Patients may thus experience a worsening of symptoms, which may explain the higher mean score for such palliative care patients. Johnsen et al. found insomnia to be one of the most frequent symptoms in advanced cancer patients [2], and Rabow et al. found that sleep quality improved with outpatient palliative medicine consultations [13]. However, this was not the case in our study. On the contrary, it seems that patients sleep quality worsened and more so at the hospital R/K. An important difference between the included hospitals were the location and duration of the initial visit. The SPC team from N/S/N did home visits that often lasted more than 1 hour, which may well have helped patients to establish a closer, more trusting relationship with the team during the first consultation. This resulted in a more comprehensive symptom assessment. Getting a chance to know the team and establish a relationship along with being involved are some of the fundamental perspectives of open and effective communication, and represent two of the core themes in a person-centred approach to care [34]. Context is the third theme found by Kitson et al. (2013) [34]. Even though Wentlandt et al. (2016) found that home visits represent a non-institutionalized setting that allows for both privacy and socialization [10], sufficient time resource allocation and a qualified SPC team who have chosen to work within the palliative care setting and who are dedicated to offering a person-centred approach seem to be at least as important as the setting in which the consultation takes place.

In relation to communication, between 95.8 and 97.9% of the patients found, the nurses to be good at both listening and communicating, and more patients were satisfied with nurses’ abilities to listen and communicate than they were with doctors’ abilities; which was also found by Ross [35]. In our study, the nurses conducted the follow-up telephone consultations and were first in line to answer phone calls from the patients. This division of labour may also reflect the reason why nurses were perceived as available and therefore able to answer questions to a larger degree than were doctors. The very high number of patients who were satisfied with the nurses’ communication indicates that the nurses succeeded in not only open communication, but also in possessing the personal qualities such as being polite, respectful, sensitive and welcoming [34]. All those abilities should be present in establishing a therapeutic nurse/patient relationship. This appears to be crucial to ensuring and identifying patients’ holistic needs, and to their involvement in decision-making. A holistic approach is a factor that is important to patients, when health professionals provide emotional support and support patients’ lives [36].

We know from previous studies that SPC improves satisfaction with care [13, 37, 38]. In this study, we found a significant difference in satisfaction at baseline between the two groups – patients from R/K scored significantly higher, with a mean score of 65.91, as opposed to N/S/N55.83 at, *p* = 0.03. In examining the individual items, the ones representing the biggest difference are those concerning attention and information from doctors. This probably reflects the fact that the patients at N/S/N only see a doctor during their first consultation, and even though it is a home visit, follow-up is primarily conducted by the patients’ GPs and community home care nurses. In comparison, patients from R/K have follow-up consultations that are conducted by a doctor and nurse from the SPC team every 3 weeks and, in the meantime, if needed, follow-up phone calls with SPC nurses. In addition, the SPC team at N/S/N only had one doctor to cover all home visits, which meant there was very little time to respond to patients’ unscheduled enquiries. The difference in satisfaction concerning attention and information from doctors might have been more related to pressure of work rather than to personal ability. The level of staffing is critical and, as shown in Table 2, R/N had a broader multidisciplinary team than did N/S/N. To access non-medical, non-nursing members of the team, the patients needed a referral from the team doctor or nurse, which may also have meant a restriction on access to the multidisciplinary team members. The shortage of doctors was also reflected in the longer waiting time at N/S/N, compared to R/K [5]. This represents inequality in access to SPC and may be another explanation as to why patients were less satisfied at N/S/N. For those patients, the inequality is exacerbated by living in a rural area, which is characterised by a shortage of GPs as well as a lower level of income and education [39]. Other studies have shown that rural residents may be less likely to receive palliative care [40, 41]. Besides, those who do not receive palliative care are more likely to receive potentially aggressive end-of-life care, spend more days in hospital in the last part of life and die in an acute care hospital [41, 42]. One reason why the difference in satisfaction is not great may be that the SPC team is made up of professionals who have actively chosen palliation as their profession and that they are dedicated to and focused on providing a person-centred approach.

A strength of the study was that patients were included from a broad, heterogeneous area and the inclusion period proceeded for 1 year. In addition, data were collected not only at baseline but also one and 3 months later. This gave us a picture of the development over time and allowed us to reveal consequences of inequity in the SPC.

On the other hand, even though we included participants over a year, only 102 of the 705 possible participants returned the first questionnaire. This may have introduced a selection bias, as the mean residual life of included patients was longer than for those who did not return the first questionnaire. Further, both symptoms and QOL seemed to worsen in the late palliative care phase, which we may not have captured. We used questionnaires to collect data; a limitation to this approach may be that, despite being validated, answers are limited to the questions asked. We would have liked to elicit more detailed and nuanced patient perspectives on what it was in the SPC consultations that was important to them. We therefore conducted interviews in a later study.

## Conclusion

Specialist palliative care is in request for many patients during the late phase of their disease. Outpatient SPC may be organized using a wide variety of models. In this article, we describe two such models – one of which involves the first encounter in the form of a home visit, while, in the other, the first encounter takes place in a hospital-based outpatient clinic. We analysed the two models using a questionnaire design and found no significant difference in satisfaction with care, communication with health professionals or in overall quality of life. In fact, it seems more important that patients have access to SPC than which model is used. Furthermore, patient participation and involvement in care seem to be important to level of satisfaction, and it may that the time available matters more than the context. Thus, SPC must leave room for a person-centred and individual approach concerning symptom control, psychosocial and spiritual support, as well as for practical issues.

## Data Availability

The datasets used and/or analysed during the current study are available from the corresponding author upon reasonable request.

## Abbreviations

CPWQ-com: Cancer Patient’s World Questionnaire – communication
EORTC QLQ-C15-PAL: European Organization for Research and Treatment of Cancer Quality of Life Questionnaire Core 15 - Palliative Care
FAMCARE: Family satisfaction
GP: General practitioner
N/S/N: Naestved/Slagelse/Nykoebing F
QOL: Quality of life
R/K: Roskilde/Koege
SD: Standard deviation
SE: Standard error
SPC: Specialist palliative care
